# The Western Dietary Pattern Combined with Vancomycin-Mediated Changes to the Gut Microbiome Exacerbates Colitis Severity and Colon Tumorigenesis

**DOI:** 10.3390/nu13030881

**Published:** 2021-03-09

**Authors:** Niklas D. Aardema, Daphne M. Rodriguez, Arnaud J. Van Wettere, Abby D. Benninghoff, Korry J. Hintze

**Affiliations:** 1 Department of Nutrition, Dietetics and Food Sciences, 8700 Old Main Hill, Utah State University, Logan, UT 84322, USA; naardema93@gmail.com; 2 Department of Animal, Dairy and Veterinary Sciences, 4815 Old Main Hill, Utah State University, Logan, UT 84322, USA; daphne.rodriguez@aggiemail.usu.edu (D.M.R.); arnaud.vanwettere@usu.edu (A.J.V.W.); 3 USTAR Applied Nutrition Research, 9815 Old Main Hill, Utah State University, Logan, UT 84322, USA

**Keywords:** vancomycin, Western diet, colitis, inflammation, colorectal cancer, bacteria, gut microbiome

## Abstract

Previous work by our group using a mouse model of inflammation-associated colorectal cancer (CAC) showed that the total Western diet (TWD) promoted colon tumor development. Others have also shown that vancomycin-mediated changes to the gut microbiome increased colorectal cancer (CRC). Therefore, the objective of this study was to determine the impact of vancomycin on colon tumorigenesis in the context of a standard mouse diet or the TWD. A 2 × 2 factorial design was used, in which C57Bl/6J mice were fed either the standard AIN93G diet or TWD and with vancomycin in the drinking water or not. While both the TWD and vancomycin treatments independently increased parameters associated with gut inflammation and tumorigenesis compared to AIN93G and plain water controls, mice fed the TWD and treated with vancomycin had significantly increased tumor multiplicity and burden relative to all other treatments. Vancomycin treatment significantly decreased alpha diversity and changed the abundance of several taxa at the phylum, family, and genus levels. Conversely, basal diet had relatively minor effects on the gut microbiome composition. These results support our previous research that the TWD promotes colon tumorigenesis and suggest that vancomycin-induced changes to the gut microbiome are associated with higher tumor rates.

## 1. Introduction

The risk for colorectal cancer (CRC) is multifaceted and factors can include genetics, age, diet, inflammation, and the composition of the gut microbiome [1]. While CRC is associated with a Western lifestyle [1,2], incidence rates in non-Western countries that have adopted more Western lifestyles, such as Japan, have increased in recent years and are now similar to incidence rates for some Western countries, such as the United States, Canada, and Australia [3]. Although, genetics can play a role in the etiology of CRC, the majority (over 80%) of patients with CRC have no family history of the disease [1]. Continuing research has increased our understanding of what non-genetic factors play a role in the onset of CRC. Two interrelated, modifiable variables implicated in CRC risk are the gut microbiome composition and diet.

The gut microbiome is an important factor for intestinal health, including CRC. Differences in the gut microbiome between healthy control subjects and patients with intestinal diseases such as CRC, ulcerative colitis, or Crohn’s disease have been demonstrated in several studies [4,5,6,7,8,9]. When comparing the relative abundances of taxa present in the intestinal microbiome of healthy individuals to patients with CRC, studies have shown an increased abundance of *Fusobacterium* [10,11,12], *Enterococcaceae* [12,13], *Peptostreptococcus* [11,12], and *Erysipelotrichaceae* [12,14], and decreased *Faecalibacterium* [11,12], *Roseburia* [11,12], *Blautia* [11], and *Bifidobacterium* [11,15,16], among others. The results of these studies support the growing idea that changes to the gut microbiome, whether a cause or a consequence of CRC, are related to the disease.

Antibiotics can be useful tools for determining how certain species or communities of bacteria affect gut health by altering the composition of the intestinal microbial community [17,18,19,20,21,22,23,24,25,26]. Oral administration of the antibiotic vancomycin has been used to evaluate the impact of changes that occur in the gut microbiome in chronic disease models because it is not absorbed systemically and its effects are localized to the gut. Vancomycin treatment in pre-clinical CRC models has led to changes in the microbiome and the severity of CRC [26,27]. However, these studies have had equivocal results. In one study [26], vancomycin pre-treatment for 2–3 weeks before a dextran sodium sulfate (DSS) challenge significantly lengthened the amount of time necessary for body weight to return to normal and decreased colonic tissue rebuilding. Conversely, Tanaka and colleagues [27] reported that vancomycin decreased gut inflammation and CRC. Mice treated with vancomycin throughout the duration of the study had decreased tumor multiplicity, improved colon histopathology scores, and decreased concentrations of several inflammatory cytokines. The gut microbial compositions of the study groups were also assessed, with vancomycin causing a decrease in several bacterial taxa, including *Bacteroides*, *Prevotella*, and *Clostridium*. It is not clear why studies that have investigated vancomycin-induced changes to the microbiome, gut inflammation, and colonic mucosa have had differing results. Several factors could be involved in this discrepancy, including study protocols (pre-treatment vs. continuous antibiotic treatment), experimental diets, gender, animal origin, or initial microbiome of the study animals.

Human epidemiological data show that the Western dietary pattern promotes CRC development. In order to model the Western dietary pattern for pre-clinical studies, we developed the Total Western diet (TWD), which reflects the 50th percentile of NHANES data for American macro- and micronutrient intakes [28]. The TWD diet accounts for the intakes of both simple and complex carbohydrates, and for the overall amount of individual fatty acids consumed. These intakes, gathered from NHANES data, were translated to a rodent diet using nutrient density (mg/kcal) as the scaling measure. We have demonstrated that the TWD promotes CRC in several different pre-clinical models. In a previous study [29], mice fed the TWD and dosed with azoxymethane (AOM) had increased aberrant crypt foci (ACF) and total crypt cells compared to controls fed the AIN93G diet. Moreover, green tea treatment reduced ACF only when administered to mice fed the TWD and not AIN93G suggesting basal diet is an important factor when assessing the bioactivity of nutraceuticals in pre-clinical chronic disease models. In a series of studies using the AOM/dextran sodium sulfate (DSS) and the APC*^min+/−^* CRC model, Benninghoff et al. [30] demonstrated that mice fed the TWD had increased colonic expression of inflammatory genes, increased colitis, and increased tumor burden relative to mice fed the AIN93G diet and that the disease phenotype could be partially rescued by increasing the calcium and vitamin D levels of the TWD. These studies suggest that the American dietary pattern promotes CRC through an increased expression of inflammatory genes in the colonic mucosa resulting in gut inflammation and CRC.

As CRC cancer can be influenced by a number of factors including the gut microbiome and diet, it is important to understand how these factors may interact in terms of CRC risk. Antibiotics, such as vancomycin, elicit a change in the overall composition of the gut microbial community, and such changes can also be associated with changes in the likelihood of CRC development. Dietary patterns, specifically a Western diet, are strongly associated with CRC. Although many studies have investigated the role of the Western diet or the gut microbiome on CRC independently, research on the intersection of the gut microbiome and the Western diet and their combined effects on CRC is limited. Therefore, in this study, both factors were examined simultaneously in order to determine the effects of the Western dietary pattern and vancomycin-induced changes to the gut microbiome on CRC.

## 2. Materials and Methods

### 2.1. Chemicals and Reagents

Azoxymethane (AOM) was obtained from Sigma-Aldrich (St. Louis, MO, USA; CAS No. 25843-45-2). Dextran sodium sulfate (DSS; reagent grade at mol. wt. ~40 kDa) was purchased from Alfa Aezar (VWR, Radnor, PA, USA). All other chemicals were obtained from general laboratory suppliers at reagent grade quality. Other reagents and kits are noted below.

### 2.2. Animals and Experimental Diets

The Utah State University Institutional Animal Care and Use Committee approved all procedures for the handling and treatment of mice used in this study (protocol 2404). Animals were housed in the Laboratory Animal Research Center (LARC) at Utah State University, which is an AAALAC approved facility. Mice were provided Bed-o’Cobs^®^ 1/4 bedding (Andersons, Cincinnati, OH, USA) and housed in HEPA-filtered cages on a IVC Air Handling Solutions ventilated housing system (Tecniplast, Buguggiate, Italy).

To be consistent with previous work on the role of the TWD in CRC (97), male C57Bl/6J mice (total *N* = 144) were purchased from Jackson Laboratories (Bar Harbor, ME, USA) at five weeks of age. Following one week of quarantine, mice were randomized and placed into one of four intervention groups (*n* = 36 per group). Mice were group-housed, four animals per cage, nine cages per intervention, in HEPA-filtered microisolator cages with Bed-o’Cobs^®^ 1/4 bedding (Andersons, Cincinnati, OH) on a IVC Air Handling Solutions ventilated housing system (Tecniplast, Buguggiate, Italy). Mice were maintained at 18 to 23 °C with humidity at 50% and with a 12:12 h dark:light cycle in the Utah State University Utah Science Technology and Research (USTAR) BioInnovation center’s specific pathogen-free vivarium. Ear notching was performed to allow for repeated individual mouse weight measurements, as mice were group-housed.

### 2.3. Study Design

To investigate the effects of the Western dietary pattern and vancomycin-mediated changes to the gut microbiome on colitis and CRC, a 2 × 2 factorial study design was used with *experimental diet* and *antibiotic treatment* as the two main factors. Experimental diets were formulated by Envigo (Hackensack, NJ, USA), obtained as a single lot from the vendor, and maintained at 4 °C for the duration of the study. Mice were fed ad libitum either a control diet (AIN93G, cat. no. TD.160421) or the TWD (cat. no. TD.160422; formulation previously published [28]) to emulate the Western dietary pattern (Table S1). The energy densities of these diets differ, with AIN93G (AIN) at 3.8 kcal/g and the TWD at 4.4 kcal/g. Half the mice from each dietary treatment were administered vancomycin at 500 mg/mL (Alvogen, Pine Brook, NJ, USA) via drinking water, while the other half were provided plain water. Mice were fed experimental diets and treated with or without vancomycin for the duration of the study. This combination of the diet and antibiotic treatment resulted in four experimental groups: AIN+Water (AIN/Wa), AIN+Vancomycin (AIN/VM), TWD+Water (TWD/Wa), and TWD+Vancomycin (TWD/VM). Fresh food was provided twice per week, and food consumption was monitored at each change (including accounting for spillage into the cage). Fresh water was provided every other day, and water intake was estimated by differential weight of the container at each water change. Individual body weights were recorded once per week for all mice.

### 2.4. Colorectal Cancer Initiation via AOM+DSS Protocol

After 14 days of experimental diets, mice were dosed *i.p.* with 10 mg/kg of AOM prepared in sterile PBS and provided 1% (*w*/*v*) DSS, a colonic irritant, via their drinking water for 10 days followed by regular tap water, with or without vancomycin depending on the experimental group, for the remainder of the study. On study day 24, when DSS treatment was complete, and again on day 38 during recovery from colitis, all mice were assessed in a blinded fashion for the colitis disease activity index (DAI) as previously described [30]. Briefly, this assessment ranks four categories: percent body weight change, stool consistency, blood in stool, and rectal bleeding on a scale of zero to four. Approximately 12% of mice died from exposure to AOM/DSS before the end of the study.

### 2.5. Colon Histopathology

On day 38 (recovery) and day 98 (terminal), subsets of mice (*n* = 12/group for recovery; *n* = 8/group for terminal) were randomly selected for histopathological assessment and colons were processed as previously described [30]. Colon tissue samples were blinded as to treatment and then assessed by a board-certified veterinary pathologist for inflammation and mucosal injury using a grading scheme previously described [31,32] with a minimum of 15 high power fields (400×, 2.37 mm^2^) examined per tissue sample.

### 2.6. Assessment of Tumors of the Colon

At the end of the study (day 98), all remaining mice were euthanized by CO_2_ asphyxiation and necropsied. During necropsy, the liver, cecum, gonadal fat, and blood plasma tissues were collected and stored for further assessment. Colons were collected, blinded to treatment, and examined for the tumors as previously described [30]. Colon length was also measured as the distance from the tumor to the end of the rectum using digital calipers. As noted above, eight colons from each of the four groups were randomly selected prior to necropsy to be evaluated for histopathology. The tumors from these colons were counted and measured immediately following necropsy, then prepared for histopathology analyses as described above.

### 2.7. Fecal Microbiome Composition and Analysis

Taxonomic measures of the fecal microbiome at the study end (day 98) were performed using 16S rRNA sequencing as described previously [33]. Bacterial DNA from the fecal samples was extracted using the QIAGEN QIAamp DNA Stool Mini Kit (Hilden, Germany) according to the manufacturer’s instructions. After DNA extraction, samples were analyzed by spectroscopy to determine the concentration of DNA for each sample and then diluted with TE buffer to a concentration of 1 ng/µL. Samples were amplified via PCR using barcoded primers directed against the V3 region of the 16S rRNA (123). PCR amplification was performed using the following protocol: 5 min at 95 °C; 35 cycles of 94 °C for 30 s, 55 °C for 30 s and 72 °C for 90 s; final annealing at 72 °C for 10 min; hold at 4 °C. Following PCR amplification, gel electrophoresis was performed confirm the presence and expected size of the amplicons. The PCR products were then purified using Agencourt AMPure microbeads (Beckman Coulter, Indianapolis, IN, USA). PCR products were washed with ethanol to eliminate excess primers, nucleotides, and enzymes present in the PCR mix, and then DNA was eluted from the beads with TE bovver. DNA concentration was measured using the Quant-iT Picogreen dsDNA assay (Thermo Fisher Scientific, Waltham, MA, USA). Samples were then diluted to 1 ng/µL in TE buffer, pooled and stored at −80 °C. Sequencing was performed at the Utah State Center for Integrated Biotechnology core sequencing facility using the Ion Personal Genome Machine (PGM) Sequencer with a 318 Chip kit and an Ion PGM Hi-Q View OT2 kit for library preparation (Thermo Fisher Scientific using the Ion Reporter™ workflow.

Sequences were processed with the latest version of QIIME [34]. Sequences were filtered for quality and assigned operational taxonomic units (OTUs) [35] at a 97% sequence similarity as compared to a reference GreenGenes OTU database (gg_13_8_otus). Sequences were assigned using the open-reference OTU picking methodology with UCLUST using pick_open_ref_otus.py workflow script [36]. Sequences at the highest levels of abundance were chosen as representative sequences, and these were checked for chimera artifacts using uchime61 [37]. The resulting .biom, metadata, and phylogenetic tree files were uploaded to the Microbiome Analyst Marker Data Profiling module for analysis [38,39]. Note that GreenGenes database incorrectly assigned the genus *Sutterella* to the family Alcaligenaceae, rather than the up-to-date classification of Sutterellaceae, which is used in this paper. The sequencing .biom file and .txt mapping file with sample annotations are publicly available [40].

Features were filtered for a minimum count of 4 with prevalence in 20% of samples, and a low variance filter was applied to remove 10% of samples based on the inter-quartile range. Data were rarefied to the minimum library size and scaled using total sum scaling. Taxonomic plots were generated using the relative abundance for the top 15 taxa. Heat tree plots were generated to visualize differences in median abundance at the species taxonomy level for specific comparisons of interests with a Wilcoxon *p*-value < 0.05. Alpha diversity was determined using the observed OTUs, chao1, and Shannon index diversity measures at the feature (OTU) taxonomic level. Principal coordinate analyses were performed using the unweighted and weighted unifrac distance measures at the feature (OUT) taxonomic level with the permutational MANOVA (permanova) statistical method. MetagenomeSeq was performed at the family and genus taxa levels to identify statistical differences in normalized abundance, with the zero-inflated gaussian fit statistical model and FDR-adjusted *p*-value < 0.05. Finally, linear discriminate analysis with effect size (lefse) was performed for each diet group at the genus level with a limit log_10_ LDA score < 2.0 and FDR-adjusted *p*-value < 0.05.

### 2.8. Other Data Analyses

Data were analyzed using a linear mixed model (*mixed* procedure) for the main effects of diet, antibiotic, and diet*antibiotic with the mouse cage as a nested, random factor (as appropriate) using the restricted maximum likelihood (REML) estimation and the Tukey HSD post hoc test for multiple comparisons (SAS vers. 9, SAS Institute Inc., Cary, NC, USA). Data that did not meet the equal variance assumption were log_10_, square-root, or cube-root transformed. Statistical analyses of survival and tumor incidence were performed using count data (alive vs. dead; normal vs. tumor) with the Fisher’s exact test followed by the Bonferroni adjustment for multiple testing among treatment groups. For all analyses, a significant effect of the test variable was inferred when the adjusted *p* value was <0.05.

## 3. Results

### 3.1. Food and Energy Intakes and Body Weight Gain

**Figure 1:**
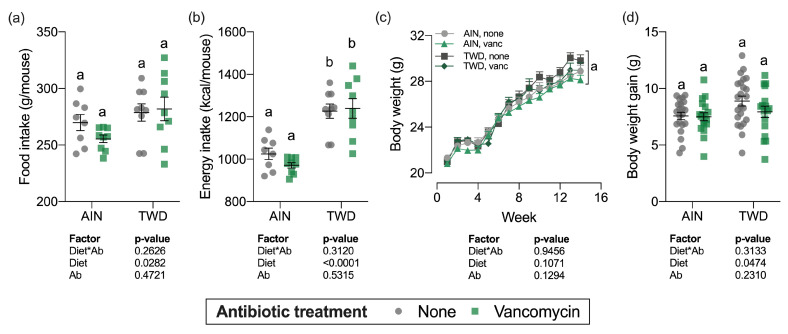
Food and energy intake and body weight gain over the study period. Total estimated food intake (**a**) and total estimated energy intake (**b**) per cage per mouse are shown as individual measurements with the mean ± standard error (*n* = 8 to 9 cages). Body weight measurements weekly (**c**) and body weight gain (differential of day 98 and day 0 weights) (**d**) are shown as mean ± standard error (**c**) or individual measurements with the mean ± standard error (**d**) (initial *n* = 36/group, final *n* = 17 to 23/group). Main effects of experimental factors diet, antibiotic treatment and diet*antibiotic interaction are shown as determined by generalized linear mixed model, and different letters indicate experimental groups are significantly different (*p* < 0.05) as determined by the Tukey HSD post hoc test (for terminal time point only for panel c). Abbreviations: AIN, the AIN93G diet; TWD, the total Western diet; Ab, antibiotic; vanc, vancomycin.

Over the course of the study, total food intake was marginally higher for mice fed the TWD compared to AIN93G fed mice (diet main effect, *p* = 0.03), which translated into a substantial increase in energy intake (diet main effect, *p* < 0.0001) owing to the higher energy density of the TWD (Figure 1a,b). However, vancomycin treatment did not affect total food or energy intake for mice fed either diet, nor was there an interaction between diet and vancomycin treatment.

Body weights of individual mice were measured weekly (Figure 1c) and total weight gain was calculated by subtracting initial body weights from final body weights (Figure 1d). As anticipated, during the DSS treatment and shortly after during active colitis, body weight gain slowed or stopped altogether (week 2–4), but soon recovered as animals recovered from the gut injury. Experimental treatment did not significantly affect the final body weights (Figure 1c), although body weight gain was marginally increased for the TWD-fed mice (diet main effect, *p* = 0.0474) (Figure 1d). For the overall study period, body weight gain or TWD-fed mice with no vancomycin was slightly higher than the other groups (Figure S1), though the final body weight for this group was not statistically different at the study end (Figure 1c).

### 3.2. Disease Activity Index and Histopathology of Inflammation and Mucosa Injury

**Figure 2:**
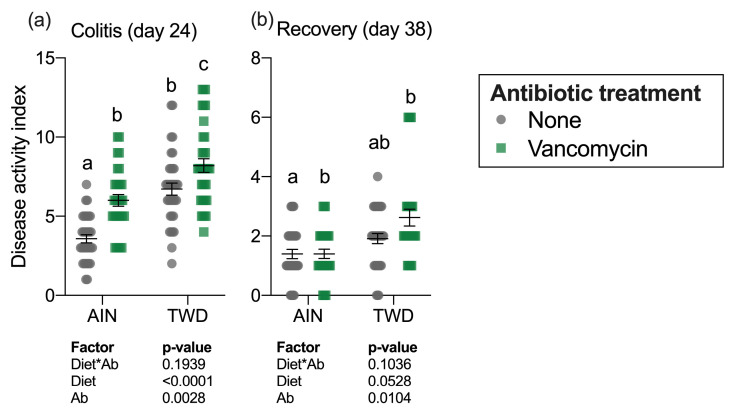
Disease activity index during active colitis (day 24) (**a**) and recovery from gut injury (day 38) (**b**). Values shown are the individual measurements for each animal with the mean ± standard error (*n* = 30 to 36). Main effects of experimental factors diet, antibiotic treatment and diet*antibiotic interaction are shown as determined by generalized linear mixed model, and different letters indicate experimental groups are significantly different (*p* < 0.05) as determined by the Tukey HSD post-hoc test. Abbreviations: AIN, the AIN93G diet; TWD, the total Western diet; Ab, antibiotic.

The colitis DAI was assessed on day 24 during active colitis immediately following DSS exposure and 14 days later on day 38 during recovery from gut injury (Figure 2a). Significant main effects of both diet (*p* < 0.0001) and vancomycin (*p* = 0.0028) were noted at the first time point, with the TWD diet markedly exacerbating colitis symptoms as has been observed repeatedly in this model [30]. Interestingly, vancomycin exposure compounded this effect, with the most severe colitis symptoms noted for mice fed TWD with vancomycin. Conversely, mice fed AIN with no antibiotic had the least severe colitis symptoms. By the recovery time point, the influence of diet on the DAI had abated, with antibiotic treatment still remaining a significant main effect (*p* = 0.0104), most notably in mice fed TWD (Figure 2b).

**Figure 3:**
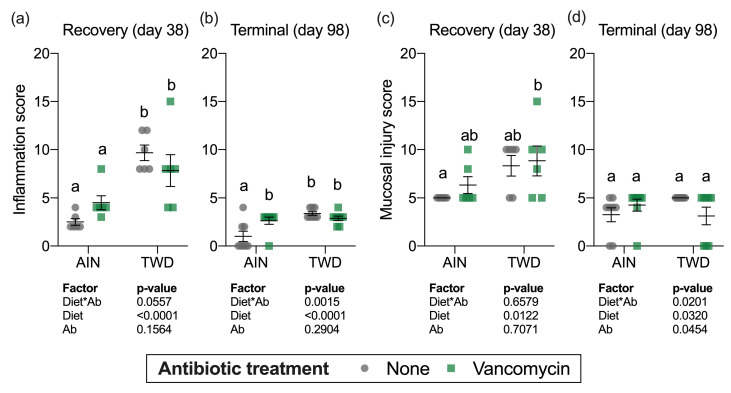
Histopathological assessment of mucosal inflammation (**a**,**b**) and mucosal injury (**c**,**d**). Values shown are the individual measurements for each animal with the mean ± standard error (*n* = 6 for day 38, *n* = 8 for day 98). Main effects of experimental factors diet, antibiotic treatment and diet*antibiotic interaction are shown as determined by generalized linear mixed model, and different letters indicate experimental groups are significantly different (*p* < 0.05) as determined by the Tukey HSD post hoc test. Abbreviations: AIN, the AIN93G diet; TWD, the total Western diet; Ab, antibiotic.

Histopathology analyses were performed on a subset of mice to determine colonic inflammation and mucosal injury scores for each of the treatment groups at the recovery timepoint (day 38) and at the study termination (day 98) (Figure 3). Similar to colitis disease activity, mice fed the TWD had increased colonic inflammation (main effect of diet, *p* < 0.0001, Figure 3a) and significant mucosal injury (main effect of diet, *p* = 0.0122, Figure 3c) with histological analyses revealing severe multifocal neutrophilic infiltration, extensive crypt and surface epithelium loss and poor tissue regeneration (*p* < 0.0001). However, vancomycin treatment did not appear to exacerbate inflammation or mucosa injury at this recovery time point (main effect of vancomycin, *p* = 0.1939 and *p* = 0.7071 for inflammation and mucosa injury scores, respectively). Moreover, no significant diet*antibiotic interactions were noted.

At the study termination, mice fed the TWD had higher inflammation scores relative to AIN-fed mice (main effect of diet, *p* < 0.0001) and, similar to the recovery timepoint, vancomycin treatment had no effect on the inflammation score (main effect of antibiotic, *p* = 0.2904) (Figure 3b). However, a significant interaction for diet*antibiotic pointed to a differential effect of vancomycin depending on the experimental diet, evident as a significant difference between control and antibiotic-exposed mice fed the AIN diet (Figure 3b). For mucosal injury, no differences among the experimental groups were noted by the study end (Figure 3d). These results suggest that the TWD has long-lasting effects on colon inflammation even after chemical-induced damage to the epithelium had ceased and mucosal injury had largely resolved, whereas vancomycin had more transient effects only increasing disease symptoms during active disease.

### 3.3. Colon Tumorigenesis

**Figure 4:**
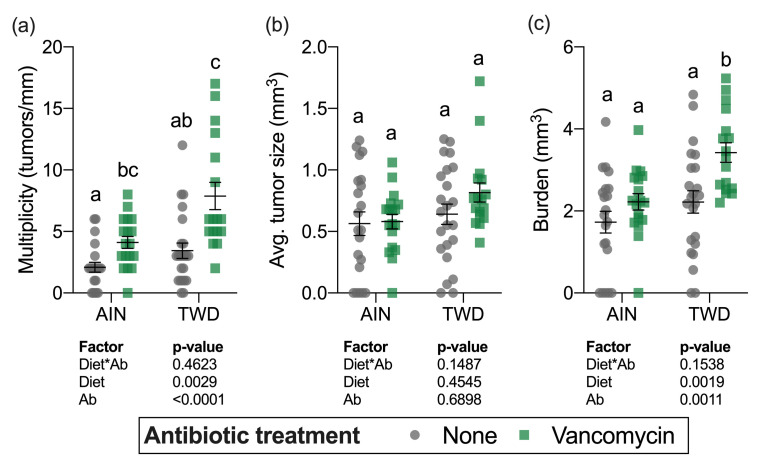
Tumor multiplicity (**a**), average tumor size (**b**) and tumor burden (**c**). Multiplicity was measured as the number of tumors per mouse normalized to the colon length. Tumor burden was calculated as the total tumor volume for each mouse. Values shown are the individual measurements for each animal with the mean ± standard error (*n* = 17 to 23). Main effects of experimental factors diet, antibiotic treatment and diet*antibiotic interaction are shown as determined by generalized linear mixed model, and different letters indicate experimental groups are significantly different (*p* < 0.05) as determined by the Tukey HSD post hoc test. Abbreviations: AIN, the AIN93G diet; TWD, the total Western diet; Ab, antibiotic.

Although a sizable fraction of mice in some treatment groups (up to 30% in mice fed TWD with vancomycin) did not survive to the study end, there was not a significant effect of treatment on overall survival after the multiple testing correction was applied (Figure S2a). At the study termination, colons were assessed for the presence (incidence), the number (multiplicity), size (volume), and total volume of tumors (burden) in mice from all experimental groups. Tumor incidence was not significantly different among the treatment groups (Figure S2b). Significant main effects of both diet and antibiotic treatment were evident for both tumor multiplicity and tumor burden (Figure 4a,c). As has been previously observed in this model [30], mice fed the TWD had a greater number of tumors and higher tumor burden (main effect of diet, *p* = 0.0019 and *p* = 0.0017, respectively). Interestingly, combining TWD with vancomycin significantly exacerbated tumor multiplicity by 2-fold (*p* = 0.0017) and tumor burden by 2.3-fold (*p* = 0.0241) compared to TWD-fed mice with no antibiotic treatment (Figure 4a,c). The average tumor size was unaffected by either diet (main effect *p* = 0.4545) or vancomycin treatment (main effect *p* = 0.6898) (Figure 4c).

### 3.4. Microbiome Taxonomy and Relative Abundance

A total of 1.1 × 10^7^ amplicons were sequenced, and after length, quality and abundance filtering, and chimera checking, 3.7 × 10^6^ sequences were assigned to OTUs using the pick_open_ref_otus command for an average of 46,727 sequences per sample assigned to 2251 OTUs. The sequencing depth for diversity analyses was set to ~16,500 sequences when including all samples and ~26,500 sequences for comparing highest and lowest tumor burden quartiles (Figure S3).

**Figure 5:**
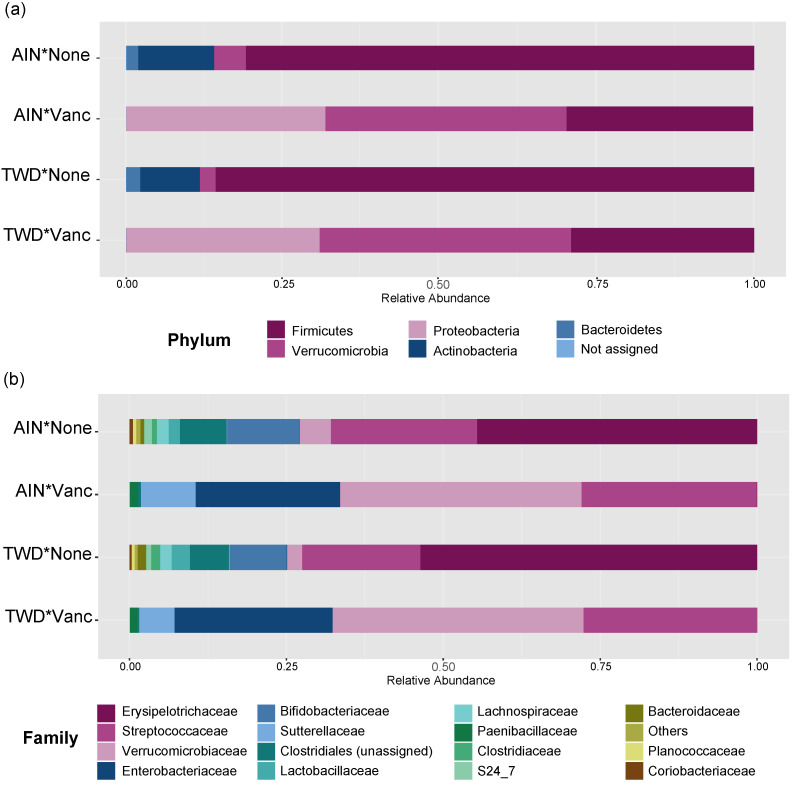
Taxonomic classification of mouse fecal bacteria. Data shown are the relative normalized abundance of bacteria annotated to phyla (**a**) or family (**b**) taxonomic levels for the top 15 most abundant taxa for each experimental group (*n* = 17 to 23). Taxonomic classifications for individual animals are provided in supplemental Figure S4. Abbreviations: AIN, the AIN93G diet; TWD, the total Western diet; Vanc, vancomycin.

**Figure 6:**
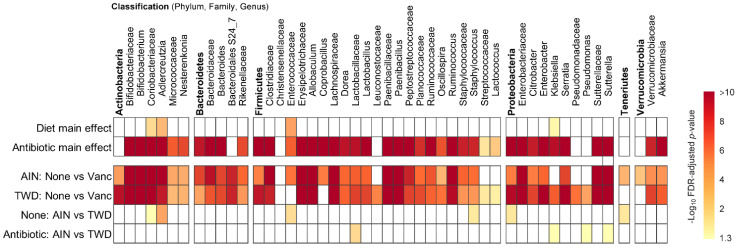
MetagenomeSeq of gut bacteria at the phylum, family, and genus taxonomic levels for main effects of diet or antibiotic and then pairwise tests for effect of antibiotic within each diet group or for diet within each antibiotic group. Values are the −log_10_ FDR-adjusted *p*-values; blank cells were not significant. Statistical results with specific *p*-values are available in Table S2. Abbreviations: AIN, the AIN93G diet; TWD, the total Western diet; Vanc, vancomycin.

Vancomycin treatment drastically changed the microbiome composition, whereas the basal diet appeared to have minor effects on a few select taxa (Figure 5 and Figure 6, Figure S3 and Table S2). At the phylum level, antibiotic treatment significantly decreased the relative abundance of Firmicutes in the fecal microbiome from approximately 80% to about 30% (main effect *p* = 2.6 × 10^−6^) and Actinobacteria from about 12% to not detected (main effect *p* = 5.61 × 10^−14^) (Figure 5a). Similarly, abundance of Bacteroidetes was also decreased from about 2% to not detected in antibiotic-exposed animals (*p* = 1.08 × 10^−9^). Conversely, vancomycin increased the relative abundance of Proteobacteria from less than 1% in control mice to about 31% in antibiotic-treated mice (*p* = 4.47 × 10^−17^). Additionally, relative abundance of *Akkermansia mucinphilla*, the single taxa representing the Verrucomicrobia phylum, was markedly increased in the feces of antibiotic-exposed mice representing nearly 40% of the microbiome compared to only 2 to 5% in control mice (*p* = 1.23 × 10^−10^).

**Figure 7:**
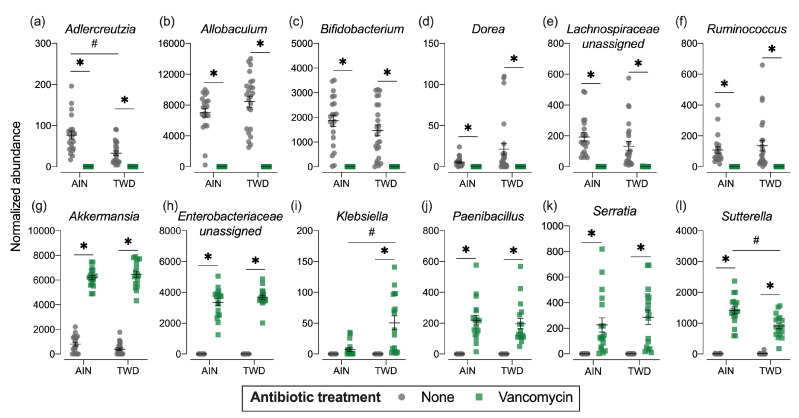
Normalized abundance of selected microbiota taxa at the family or genus level for experimental groups. Values shown are the individual measurements for each animal with the mean ± standard error (*n* = 17 to 23). * indicates a significant effect of vancomycin (FDR-adjusted *p* < 0.05) compared to untreated control within each diet group; # indicates a significant effect of diet (FDR-adjusted *p* < 0.05) comparing TWD to AIN diet within each antibiotic group as determined by metagenomeSeq using a zero-inflated gaussian fit statistical model. See Table S2 for specific *p*-values for pairwise comparisons and for other taxa. Abbreviations: AIN, the AIN93G diet; TWD, the total Western diet; Ab, antibiotic.

Phylogenetic heat trees shown in Figure S5 illustrate differences in relative abundance of bacteria at all taxonomic levels for the whole microbiome for the four pairwise comparisons of primary interest: comparing the effect of vancomycin within each diet group and comparing the effect of diet for vancomycin or control mice. Exploration of bacteria abundance at family and genera taxonomic levels provides greater resolution for the effects of either the basal diet or antibiotic treatment on microbiome composition. Key taxa with marked decrease in relative abundance in mice treated with vancomycin include *Allobaculum* (family Erysipelotrichidae), *Bifidobacterium* (family Bifidobacteriacea)*, Ruminococcus* (family Ruminococcaceae), *Dorea* (family Lachnospiraceae), and other unassigned *Lachnospiraceae* taxa (Figure 7b–f and Figure S4a,b). However, no significant effects of basal diet were evident for any of these particular genera. Conversely, the relative abundance of *Akkermansia* (as noted above), *Klebsiella* (family Proteobacteria), *Paenibacillus* (family Paenibacillaceae), *Serratia* (family Enterobacteriaceae) and *Sutterella* (family Sutterellaceae) as well as unassigned members of the Enterobacteriaceae family were markedly increased in mice provided vancomycin (Figure 7g–l and Figure S4a,b). While most changes in bacteria abundance in response to the antibiotic treatment were consistent with respect to the basal diet, some interactions were evident suggesting a differential effect of vancomycin in the context of the Western diet (Figure S5c,d). For example, the relative abundance of *Adlercreutzia* (family Coriobacteriacea) was lower in mice fed the TWD compared to the AIN diet in the absence of antibiotic treatment (*p* = 2.91 × 10^−4^), while vancomycin eradicated this genus altogether in mice consuming either diet (*p* = 1.46 × 10^−13^ for AIN diet and = 1.70 × 10^−9^ for TWD diet) (Figure 7a and Figure S4c,d). In response to vancomycin, *Sutterella* spp. were significantly more elevated in mice fed the AIN diet as compared to those fed TWD (*p* = 0.0455) (Figure 7l), while the opposite pattern was noted for *Klebsiella* spp. (*p* = 0.0455) (Figure 7i).

Phylogenetic heat trees shown in Figure S5 illustrate differences in relative abundance of bacteria at all taxonomic levels for the whole microbiome for the four pairwise comparisons of primary interest: comparing the effect of vancomycin within each diet group and comparing the effect of diet for vancomycin or control mice. Exploration of bacteria abundance at family and genera taxonomic levels provides greater resolution for the effects of either the basal diet or antibiotic treatment on microbiome composition.

### 3.5. Alpha and Beta Diversity

**Figure 8:**
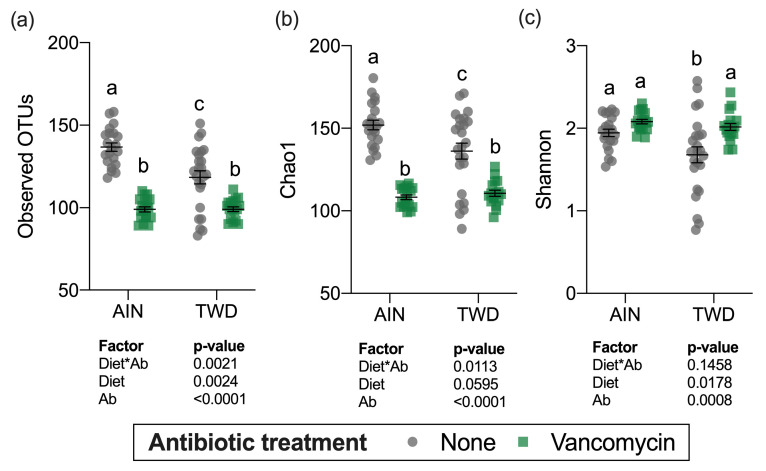
Alpha diversity of fecal microbiomes. (**a**) Observed OTUs, (**b**) Chao1 richness, and (**c**) Shannon diversity index alpha diversity values are shown are the individual measurements for each animal with the mean ± standard error (*n* = 17 to 23). Main effects of experimental factors diet, antibiotic treatment and diet*antibiotic interaction are shown as determined by generalized linear mixed model, and different letters indicate experimental groups are significantly different (*p* < 0.05) as determined by the Tukey HSD post hoc test. Abbreviations: AIN, the AIN93G diet; TWD, the total Western diet; Ab, antibiotic.

Alpha diversity was determined using three measurements: the number of observed operational taxonomic units (OTUs), the Chao1 index (count of species), and the Shannon index (accounts for proportional abundance) (Figure 8). Interestingly, all three measures of alpha diversity were significantly, though moderately decreased in mice fed the TWD compared to those provided the AIN diet in the absence of antibiotic treatment. Vancomycin treatment caused marked declines in observed OTUs and the Chao1 index, which gives weight toward rare species in mice fed either basal diet (main effect of antibiotic *p* < 0.001) (Figure 8a,b). However, the Shannon index was not altered by antibiotic treatment (Figure 8c) suggesting that the changes in diversity were most pronounced for rarer taxa.

**Figure 9:**
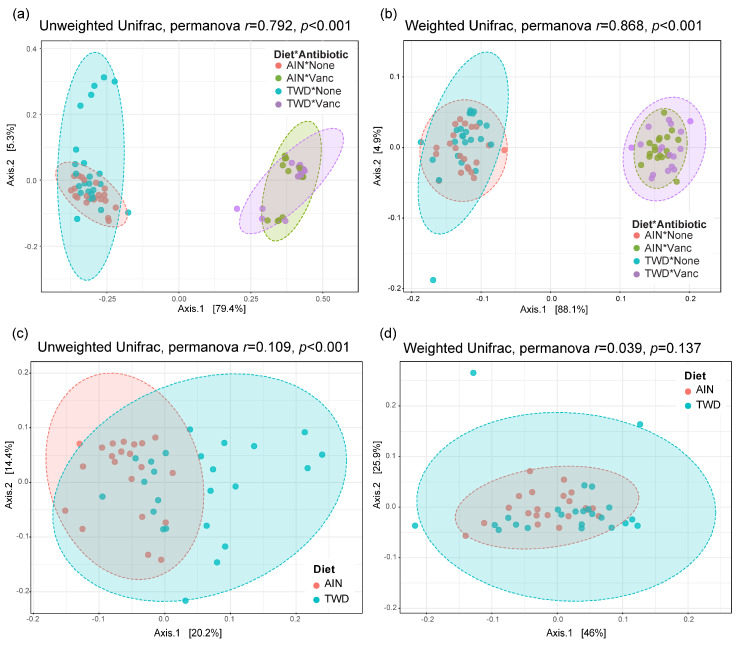
PCoA plots depicting beta diversity of fecal microbiomes. (**a**,**b**) Unweighted (**a**) and weighted (**b**) unifrac distances for all experimental groups. (**c**,**d**) Unweighted (**c**) and weighted (**d**) unifrac distances for AIN and TWD groups provided water with no antibiotic. The first two components and the variation attributed are shown, and permanova *r* and *p* values are provided for each plot. Abbreviations: PCoA, principal coordinate analysis; AIN, the AIN93G diet; TWD, the total Western diet; Ab, antibiotic.

Beta diversity unweighted and weighted unifrac distance matrices were visualized as principal coordinate plots for all four experimental groups (Figure 9a,b) and for control mice only to compare effect of basal diets (Figure 9c,d). Very clear separation by vancomycin treatment was evident for both unweighted (permanova *p* < 0.001) and weighted (*p* < 0.001) unifrac distances, indicating that the population of microbes in antibiotic-exposed mice was markedly distinct from that of control mice. Alternatively, separation by basal diet was not evident when considering all experimental groups. Although a significant difference in unweighted unifrac distance beta diversity was noted (permanova *p* < 0.001) for control mice fed either the AIN or TWD basal diets (Figure 9c), the very low *r*^2^ value of 0.109 indicates that most of the variation was not explained by basal diet. Collectively, these diversity measures suggest an outsized effect of vancomycin on gut microbiome composition that dwarfed effect of the basal diet.

### 3.6. Linear Discriminant Analysis with Effect Size

**Figure 10:**
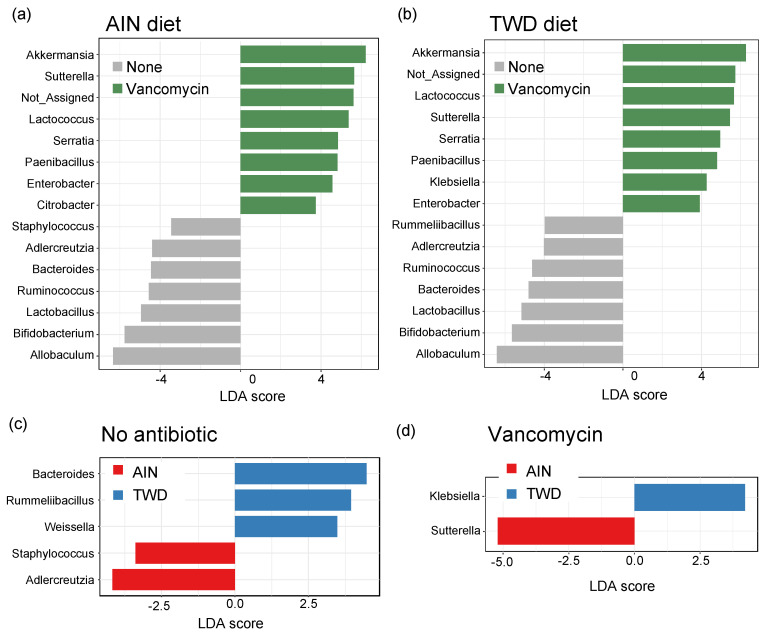
Identification of discriminating genera using linear discriminate analysis by effect size (lefse). Lefse analyses were performed to identify taxa discriminating the antibiotic treatment for mice were fed either AIN (**a**) or TWD (**b**) or to identify taxa discriminating the basal diet for no antibiotic (**c**) or vancomycin (**d**) treatment. Lefse analyses were performed at the genus taxa level with minimum score of 2.0 and FDR-adjusted *p* < 0.05. The top 15 discriminating genera are shown with the log_10_ LDA score. Abbreviations: PCoA, principal coordinate analysis; AIN, the AIN93G diet; TWD, the total Western diet.

Lefse analysis of discriminating features revealed that many of the same genera were discriminating for either control or vancomycin treatment, regardless of the basal diet (Figure 10a,b). *Akkermansia* was the strongest discriminating feature for vancomycin treatment in both diet groups, with *Lactococcus*, *Sutterella*, *Serratia*, *Paenibacillus*, and *Enterobacter* also indicating antibiotic exposure. Conversely, *Allobaculum* was the most discriminating for the control mice, with other indicator genera including *Adlercreutzia*, *Bacteroides*, *Ruminococcus*, *Lactobacillus*, and *Bifidobacterium*. Many fewer genera discriminated the basal diet, and those significant taxa differed depending on the antibiotic treatment. For control mice, *Bacteroides*, *Rummeliibacillus*, and *Weissella* genera indicated mice fed TWD compared to *Adlercreutzia* and *Staphylococcus* that discriminated mice fed the AIN diet (Figure 10c). In mice treated with vancomycin, only two discriminating taxa were noted with *Klebsiella* indicating the TWD and *Suterella* the AIN basal diet (Figure 10d).

### 3.7. Stratification of Microbiome Taxonomy by Tumor Burden Quartile

**Figure 11:**
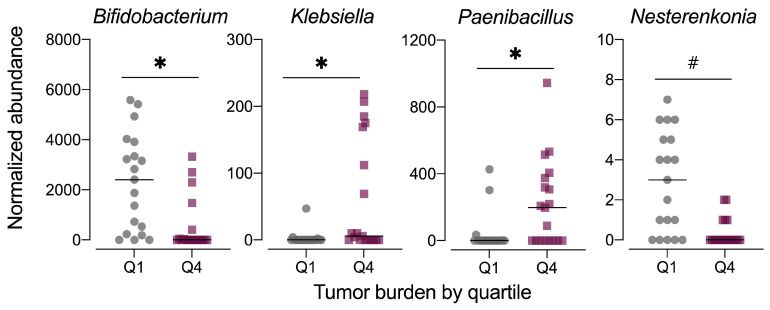
Normalized abundance of selected microbiota taxa at the genus level for samples segregated by tumor burden into quartiles. Values shown are the individual measurements for each animal in either the lowest quartile (Q1) or highest quartile (Q4) of tumor burden with the mean ± standard error (*n* = 19/quartile). # indicates *p* < 0.10 for tumor burden stratified by quartile as determined by metagenomeSeq using a zero-inflated gaussian fit statistical model. Complete results for all taxa are provided in Table S2.

As the fecal microbiome samples were obtained at the study end, the composition of the microbiome may be influenced by disease phenotype, specifically the severity of colorectal tumorigenesis triggered by the AOM+DSS protocol. Thus, we also examined the fecal microbiome data in context of tumor burden by stratifying the data set into quartiles, with quartile 1 (Q1) having the lowest tumor burden and quartile 4 (Q4) having the highest. Although the average relative abundance profiles for microbiomes at these extremes of tumor burden appeared different (Figure S6a), the data were highly variable by individual animal (Figure S4b). MetagenomeSeq revealed significant differences in the relative abundance of only three genera, including *Bifidobacterium* (family Bifidobacteriaceae), *Paenibacillus* (family Paenibacillaeceae), and *Klebsiella* (family Enterobacteriaceae), with a trend for significance for *Nesterenkonia* (family Micrococcaceae) (Figure 11, Table S2). No significant differences in alpha diversity (observed OTUs, Chao1 index, Shannon index) were noted for tumor burden-stratified data (Figure S6b). Both unweighted and weighted unifrac distance beta diversity measures were statistically significant (permanova *p* < 0.015), although the low *r*^2^ values (<0.2) indicate that most of the variation is not explained by tumor burden quartile (Figure S6c).

## 4. Discussion

Both the Western dietary pattern and the gut microbiome composition are important variables in the etiology of CRC [10,11,41,42,43,44]. However, because diet strongly influences the gut microbiome, it is difficult to disentangle how these factors independently influence CRC. We have previously shown that the TWD promotes gut inflammation and CRC [29,30], but also changes the gut microbiome relative to control AIN93 diet [33]. Yet, it is not known what role these changes to the microbiome have in relation to the increased CRC phenotype observed when mice are fed the TWD. Therefore, we investigated the effects of feeding the TWD or a control diet on colitis and CRC in mice with vancomycin altered microbiomes. We hypothesized that dietary pattern coupled with antibiotic mediated changes to the gut microbiome could modify CRC risk. As expected, vancomycin treatment elicited a dramatic change to the gut microbiome irrespective of dietary treatment as evidenced by changes in taxonomic abundances, alpha and beta diversities. Both vancomycin treatment and feeding the TWD increased gut inflammation, tumor multiplicity and burden. We did not observe a significant interaction between these factors suggesting that the Western dietary pattern and vancomycin mediated changes to the microbiome are independent risk factors for CRC.

Mice fed the TWD had increased colitis disease activity, colonic inflammation, and colon tumor burden and multiplicity. Previous work by our group with the TWD [29] has shown that in mice initiated with AOM but not given DSS, mice fed the TWD had increased colonic ACF compared to animals fed the AIN93G diet. Similarly, another group [45] using the TWD demonstrated that A/J mice fed 100 mg/kg of the foodborne carcinogen 2-amino-1-methyl-6-phenylimidazo(4,5-b)pyridine (PhIP) had increased ACF relative to mice fed the AIN93G diet suggesting that the carcinogenic phenotype elicited by feeding the TWD is not exclusive to the AOM induction model. Benninghoff et al. [30] demonstrated that mice fed the TWD had altered colon mucosa gene expression related to interferon response, inflammation, innate immunity, adaptive immunity, and antigen processing pathways relative to cohorts fed the AIN93G diet. These gene expression patterns were also accompanied by increased colitis disease activity, delayed recovery and enhanced colon tumorigenesis. This finding suggests that the TWD promotes CRC through aberrant mucosal gene expression leading to increased gut inflammation. All of these investigations, including the present study, suggest that exposure to the Western dietary pattern negatively impacts gut health and is a risk factor for CRC.

We are not the first to demonstrate antibiotic-induced gut microbial dysbiosis increases markers for CRC. For example, a study with the genetic *Apc^Min/+^* model of intestinal cancer found that mice treated with an antibiotic cocktail composed of vancomycin, neomycin, and ampicillin had increased tumorigenesis compared to control animals [46]. Mice treated with the antibiotic cocktail had significantly increased polyp size and multiplicity at 16 weeks of age compared to those not receiving antibiotics. Consistent with the current study, antibiotic treatment also increased measures of colitis disease activity, including weight loss, loose stool consistency, and blood in stool. Additionally, similar to our findings, antibiotic treatment decreased alpha diversity measures and caused alterations to relative taxa abundance. At the phylum level, Bacteroidetes and Actinobacteria were significantly decreased, while Firmicutes and Tenericutes were significantly more abundant. Abundance of the genus *Enterococcus* was significantly greater when mice were treated with antibiotics, while *Bacteroides*, *Lactobacillus*, *Roseburia*, and *Odoribacter* were all significantly less abundant. Antibiotic treatment also caused significant beta diversity clustering. These results are similar to the current study, we also observed significantly lower relative abundances of Actinobacteria and Bacteroidetes in vancomycin-treated mice. Our findings agree with many of the results of this study, suggesting that antibiotic-induced microbial dysbiosis can play a role in CRC development. However, other studies treating animals with broad spectrum antibiotic cocktails have found decreased tumor incidence as a result of treatment [47,48], suggesting that the type of antibiotic administered plays a role in determining CRC outcome.

Others have also investigated the effects of vancomycin treatment on CRC using the DSS inflammation model but the results have been equivocal [26,27]. Zhao et al. [26] reported that vancomycin treatment before DSS-induced colon inflammation increased markers of inflammation relative to non-treated controls; however, changes to the gut microbial compositions of the study populations were not reported. In a similar study, Tanaka et al. [27] reported significant decreases in the abundance of *Bacteroides/Prevotella*, *Faecalibacterium prausnitzii*, *Clostridium leptum, Clostridium coccoides,* and segmented filamentous bacteria (SFB) as result of vancomycin treatment. They also reported that vancomycin treatment decreased mucosal injury, increased colon length, and decreased tumor number. In the present study, we saw the opposite effect as vancomycin treatment increased tumorigenesis and worsened mucosal injury, gut inflammation, and colitis disease activity. Tanaka et al. also reported reduced abundance of *F. prausnitzii* as a result of vancomycin treatment, and that vancomycin treatment reduced markers for CRC [27]. However, *F. prausnitzii* is a butyrate-producing bacterium that has, in a number of studies, been shown to have anti-inflammatory properties and is protective against CRC in both animals and humans [49,50,51,52,53]. These studies all showed that lower abundance of *F. prausnitzii* was associated with an increase in CRC. In the present study, we did not find any differences in the abundance of *Faecalibacterium* in vancomycin treated mice. A number of factors may explain the equivocal results of these studies, including gender of mice, duration of vancomycin treatment, duration of DSS treatment, and importantly, the initial microbiome of experimental animals.

Compared to basal diet, vancomycin treatment had a dramatic effect on the gut microbiome. Vancomycin treatment increased relative abundance of *Akkermansia*, *Klebsiella*, and *Sutterella* irrespective of basal diet, while *Allobaculum, Bifidobacterium*, and *Dorea* were more abundant in the water treatment groups. Many of these bacteria have previously been shown to be associated with CRC. The *Akkermansia* genus has been shown to be associated with increased CRC in several animal and human studies [47,48,54,55]. One pre-clinical trial [48] induced tumorigenesis via the AOM/DSS model. This study found that *Bacteroides, Odoribacter*, and *Akkermansia* were associated with tumor growth. These results are supported by the findings of Dingemanse et al. [47], who also found that *Akkermansia muciniphila* was associated with increased tumor burden in mice. Furthermore, a study comparing the microbial populations of healthy individuals versus CRC patients found that CRC was associated with a 3.6-fold increase in *Akkermansia muciniphila* [55]. We also found that mice not treated with antibiotics had a greater shift towards *Allobaculum, Bifidobacterium*, and *Dorea* compared to vancomycin treated mice. The mice on the water only treatments also had lower tumor burden and multiplicity. Other studies have found that differences in these taxa are related to CRC [55,56]. *Dorea* has been shown to be negatively associated with CRC in a clinical study [55]. In that study, healthy individuals had a 2.9-fold greater abundance of *Dorea* present in their fecal microbiota compared to CRC patients. However, a separate clinical trial [56] found the opposite relationship, with *Dorea* being significantly more abundant in CRC patients compared to healthy controls.

In the present study, we determined that the abundance of the bacterial genera, *Bifidobacterium, Nesterenkonia, Klebsiella, and Paenibacillus* was negatively associated with tumor burden when comparing the abundances of these genera between the highest and lowest quartiles of tumor burden of all treatments. This suggests an association exists between these taxa and increased tumor burden. However, it is unclear in our study whether this is a causal relationship or whether a low tumor burden environment is favorable for these taxa. While there is limited research regarding the role of *Nesterenkonia, Klebsiella, and Paenibacillus* in CRC development and progression, *Bifidobacterium* has been well studied in relation to CRC. In a study that tested the effects of daily supplementation with *Lactobacillus acidophilus, Lactobacillus rhamnosus,* and *Bifidobacterium bifidum* using the AOM/DSS-murine model for CRC [57], it was reported that probiotic supplementation increased *Allobaculum* and *Bifidobacterium* abundance, and that probiotic consumption was negatively correlated with CRC. Another study [58] found that supplementation of the AIN76A diet with *Bifidobacterium longus* cultures reduced the number of ACF in Fisher 344 rats. A similar study reported that consumption of a probiotic containing both high and low doses of *Bifidobacterium* cultures resulted in the formation of fewer ACF compared to control animals when initiated with 1,2-dimethylhydrazine (DMH) to induce tumorigenesis [59]. High and low *Bifidobacterium* also reduced mitotic index, a marker for cell proliferation, in DMH-treated mice compared to DMH-treated controls. This study also found that high doses of *Bifidobacterium* resulted in decreased tumor multiplicity [59].

There are a few potential mechanisms by which *Bifidobacterium* may influence CRC [60,61,62,63]. One of these is through reducing the activity of enzymes harmful to gut health [61,62,63]. A second is through the inhibition of NF-κB in the intestinal epithelium thereby suppressing inflammation [60]. One study that investigated the effects of *Bifidobacterium* on CRC found that *B. adolescentis* has immunomodulatory and tumor-suppressing effects, and also reduces the activity of enzymes harmful to gut health [61]. Researchers treated cultured cells from CRC cell lines with *B. adolescentis* isolated from the feces of healthy individuals, and this inhibited cancer cell growth [61]. Increasing concentrations of *B. adolescentis* also led to increased concentrations of TNF-α, which is cytotoxic to tumor cells but also associated with increased inflammation [61]. Oral inoculation of rats with *B. adolescentis* resulted in the decreased activity of several enzymes, namely β-glucosidase, tryptophanase, β-glucuronidase, and urease [61]; these effects are potentially cancer-protective [64]. Another study of *B. adolescentis* showed similar effects on TNF-α concentrations as well as similar effects on growth of CRC cells [63]. Taken together, these studies make a case that *Bifidobacterium* may have a mechanistic role in the etiology of CRC. The results from our study align with the literature that suggests an inverse relationship between CRC and *Bifidobacterium* abundance. It is possible that in our study that *Bifidobacterium* may have played a role in the differential CRC outcome between treatment groups. However, the design of the current study did not allow us to directly test this hypothesis. Moreover, the beneficial effects of *Bifidobacterium* are strain dependent as outlined above and were unable to achieve this resolution in our microbiome analysis. Future work examining the effects of dietary pattern and strain specific *Bifidobacterium* supplementation on CRC could help determine whether *Bifidobacterium* has a causative or correlative role in our model.

Epidemiological data suggests that antibiotic treatment affects the risk for CRC. Studies have found that prolonged antibiotic use, as well as more frequent use, is associated with an increased risk for CRC [65,66]. Research has also suggested that the timing of antibiotic use may influence how great an impact they have on CRC development [67]. It has been shown that women who used antibiotics for more than 2 months between the ages of 20–39 had an odds ratio (OR) for CRC of 1.35, compared to 1.64 in women ages 40–59 with the same amount of antibiotic use [67]. While these data indicate an association between antibiotic use and CRC, several other factors may also be the cause for such a relationship. For one, a suppressed immune system as a result of malignant cancer may necessitate an increased use of antibiotics. Thus, antibiotic use would be a consequence of CRC rather than a cause. Another possible confounding factor is the high variation of dietary patterns, BMI, and gut inflammation, all of which have been shown to alter people’s risk for CRC [43,68,69,70,71,72]. However, our data suggest that long-term vancomycin treatment may be a contributing factor in CRC and consuming a Western diet may exacerbate this risk.

## 5. Conclusions

In this study, we have further demonstrated that the TWD, a diet formulated to model the typical American diet, has the significant main effects of increasing colonic inflammation and tumor development. We have also demonstrated that vancomycin-induced changes to the gut microbiome also increase colitis and CRC. The effects of vancomycin, however, have been differential among various studies, including ours [26,27]. Further investigation is needed to determine why discrepancies in pre-clinical models of vancomycin treatment and CRC exist, and what these discrepancies mean for translation of results from animal models to humans. We did not find a significant interaction between basal diet and vancomycin treatment on measures of colitis and CRC, suggesting that these two factors independently promote disease phenotype. Even though mice treated with vancomycin had very similar microbiomes regardless of basal diet, the TWD increased colitis DAI, gut inflammation, and tumor burden relative to vancomycin-treated cohorts fed the AIN93G diet. This finding suggests that increased colonic inflammation and CRC associated with feeding the TWD is independent from diet-induced changes to the microbiome. Despite this lack of an interaction, further research is warranted to examine the relationship between antibiotic use and CRC in populations consuming Western diets. Some limitations of the current study include analyzing the microbiome only at the terminal timepoint, sequencing only the V3 16s region, and use of the Ion Torrent sequencing platform and QIIME1 for amplicon processing. Future studies with multiple timepoints and analyzing the V3-V4 16s region with the latest sequencing/amplicon processing protocols may be warranted to further understand the time course of microbiome changes associated with the experimental treatments.

## Data Availability

Supporting sequencing data for this manuscript are publicly available at the Utah State University Digital Commons repository, https://doi.org/10.15142/T31D15. Available files include .txt mapping file with sample attribute information and the original .biom file with terminal fecal 16S rRNA sequencing data. Biom files are readable using QIIME software, available at Qiime.org. All other data are contained within the article and accompanying supplementary material.

## Supplementary Materials

The following are available online at https://www.mdpi.com/2072-6643/13/3/881/s1, Figure S1: Body weight gain over time for mice fed the standard AIN diet or mice fed the TWD, with and without vancomycin treatment; Figure S2: Overall survival and tumor incidence at the study end on day 98; Figure S3: Rarefaction curve analysis by experimental group; Figure S4: Taxonomic classification of mouse fecal bacteria by individual animal for all experimental groups or for the highest and lowest tumor burden quartiles; Figure S5: Phylogenetic heat trees for selected comparisons among experimental groups; Figure S6: Taxonomic classification and diversity analyses for high and low tumor burden; Table S1: Composition of experimental diets, Table S2: MetagenomeSeq analyses of differential abundance of family and genus-level taxa by experimental group and tumor burden.
