# Supplementary material for: The Western Dietary Pattern Combined with Vancomycin-Mediated Changes to the Gut Microbiome Exacerbates Colitis Severity and Colon Tumorigenesis
Source: Nutrients. 2021 Mar 9;13(3):881. doi: 10.3390/nu13030881 (PMC8000903; doi:10.3390/nu13030881)
Supplement: Supplementary file 1 [file nutrients-13-00881-s001.pdf]

nutrients

Supplementary Material

The Western dietary pattern combined with vancomycin-mediated changes to the gut microbiome exacerbates colitis severity and colon tumorigenesis

Niklas D. Aardema, Daphne M. Rodriguez, Arnaud J. Van Wettere , Abby D. Benninghoff*, Korry J. Hintze *

* Correspondence: KJH, korry.hintze@usu.edu, Tel.: +01-435-797-2124; ADB, abby.benninghoff@usu.edu, Tel.: +01-435-797-8649.

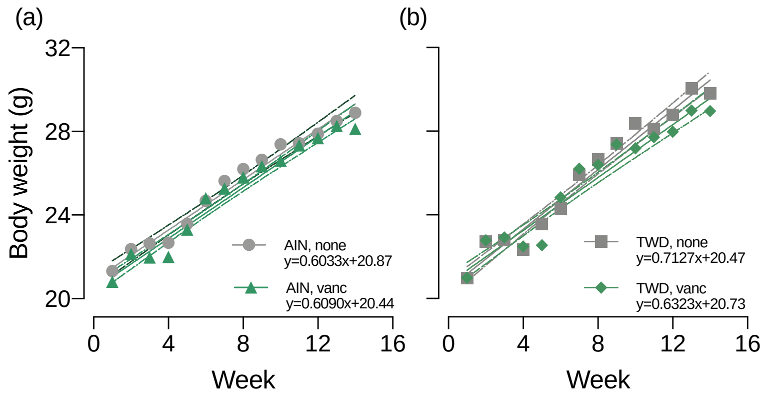

ANCOVA *p*-values

Comparison	Slope	Intercept
AIN,none vs. AIN, vanc	0.8629	0.0037
TWD, none vs. TWD, vanc	0.0360	not reported
AIN,none vs. TWD,none	0.0023	not reported
AIN,vanc vs. TWD,vanc	0.5104	0.0024

Figure S1. Body weight gain over time for (a) mice fed the standard AIN diet or (b) mice fed the TWD, with and without vancomycin treatment. The linear regression (solid line) and 95% confidence intervals are show for each experimental group. Results of analysis of covariance (ANCOVA) are shown for specific comparisons to determine if the slope and/or intercepts for regressions are statistically significant among experimental groups. Abbreviations: AIN, the AIN93G diet; TWD, the total Western diet; Ab, antibiotic.

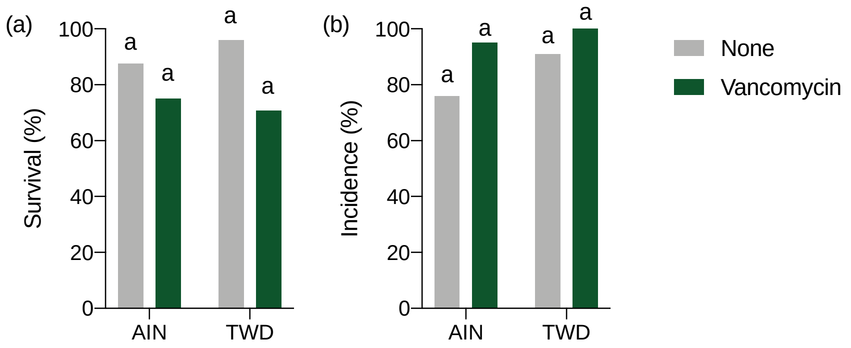

Figure S2. Overall survival (**a**) and tumor incidence (**b**) at the study end on day 98. Survival percentage shown for initial *n* = 24 per treatment group. Incidence percentage as fraction of mice with tumors for those mice that survived to day 98 (*n*=17 to 23). No significant differences in survival or tumor incidence percentage were found using the Fisher's exact test followed by the Bonferroni adjustment of *p*-values for multiple testing. Abbreviations: AIN, the AIN93G diet; TWD, the total Western diet; Ab, antibiotic.

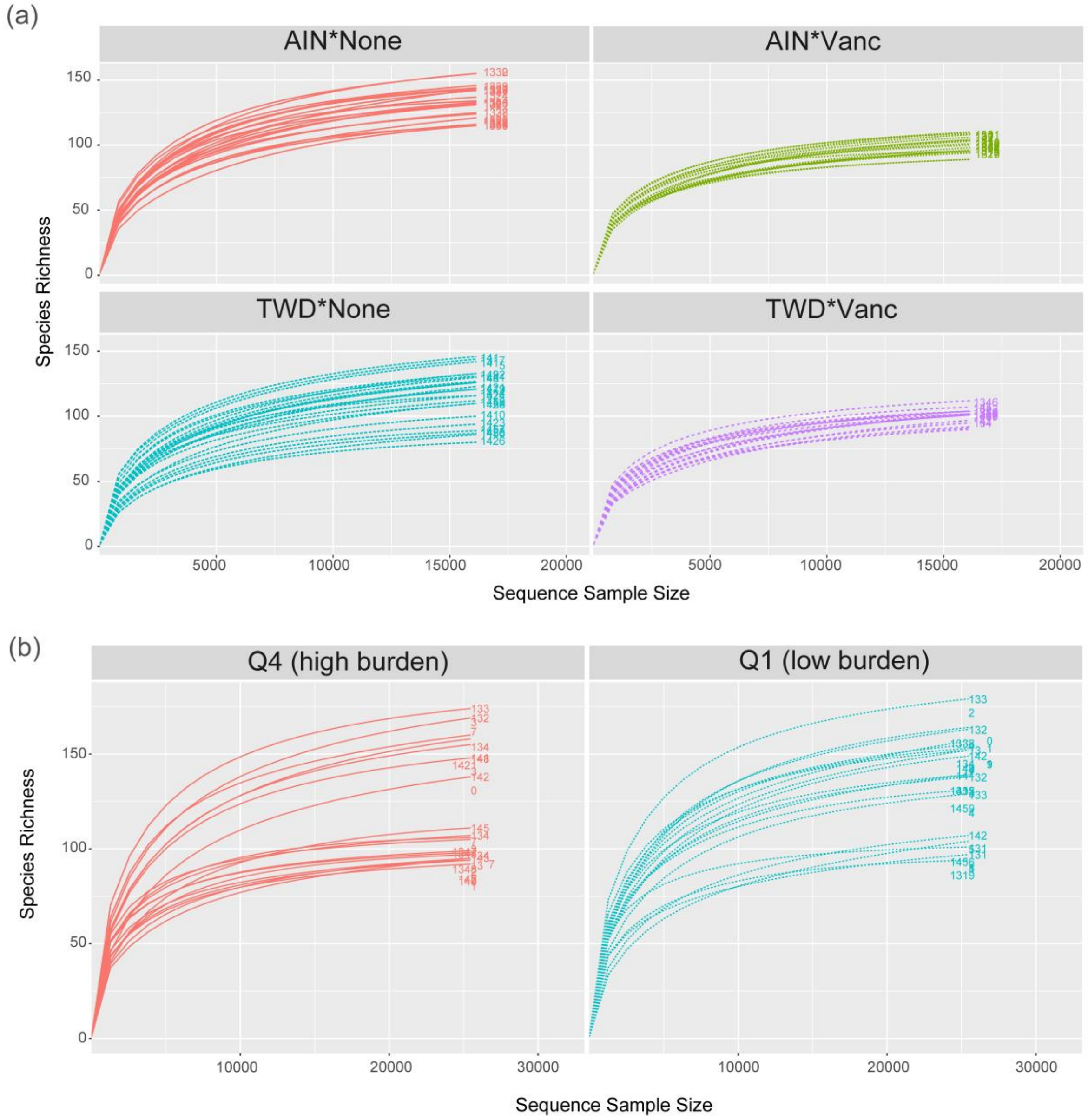

Figure S3. Rarefaction curve analysis by experimental group. Curves plot species richness as a function of sequence sample size. (**a**) For comparisons across experimental groups, data were rarefied to 16,459 sequences, the lowest total among all the samples. (**b**) For comparison of highest tumor burden (top 25%, Q4) versus lowest tumor burden (lowest 25%, Q1), rarefaction was to 26,519 sequences. These curves indicate that saturation was reached satisfactorily for the large majority of samples and that any additional sequence reads were unlikely to substantially increase the number of species detected.

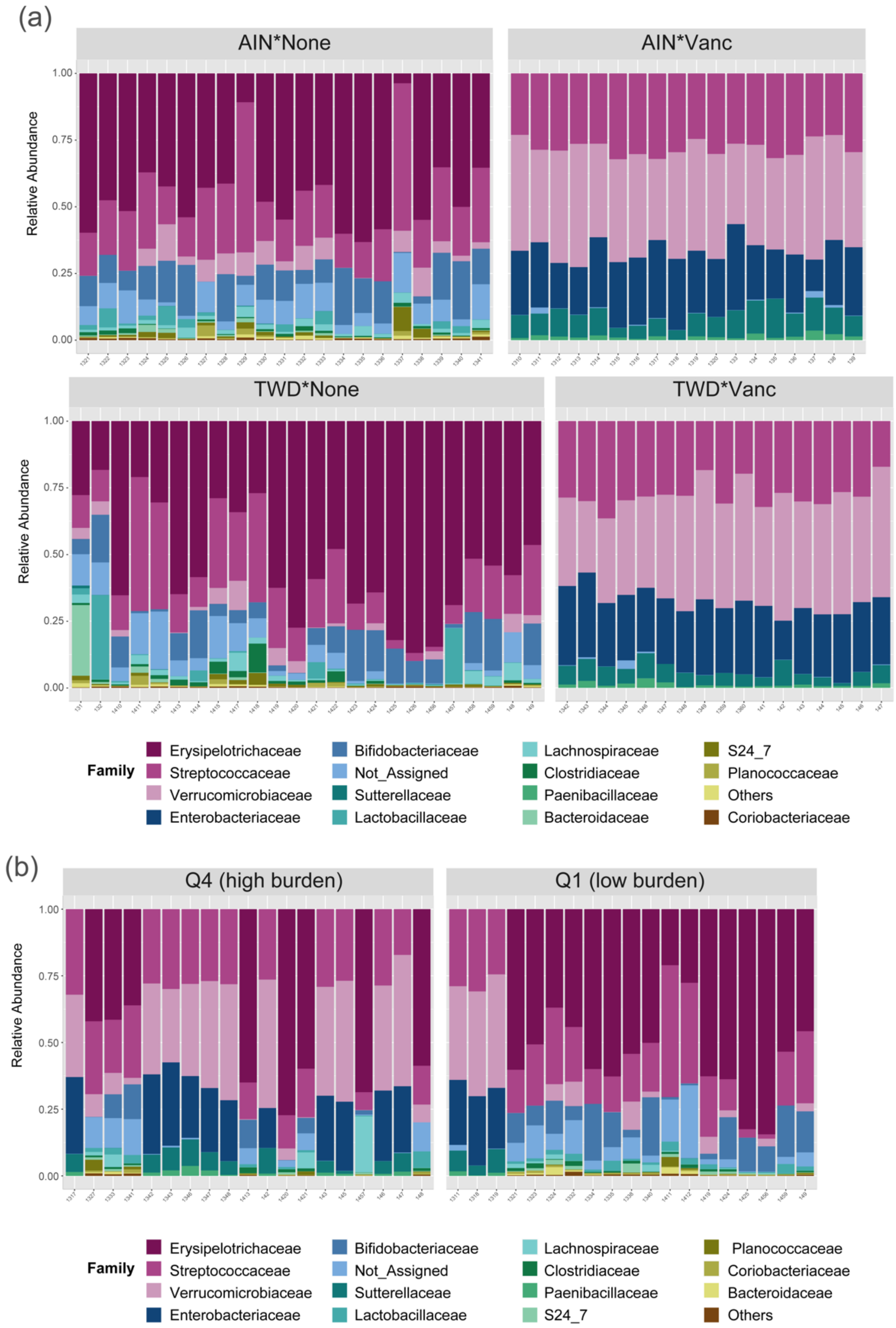

Figure S4. Taxonomic classification of mouse fecal bacteria by individual animal for all experimental groups (**a**) or for the highest and lowest tumor burden quartiles (**b**). Data shown are the relative normalized abundance of bacteria for each individual mouse annotated to the family taxonomic level for the top 15 most abundant taxa. Abbreviations: AIN, the AIN93G diet; TWD, the total Western diet; Vanc, vancomycin.

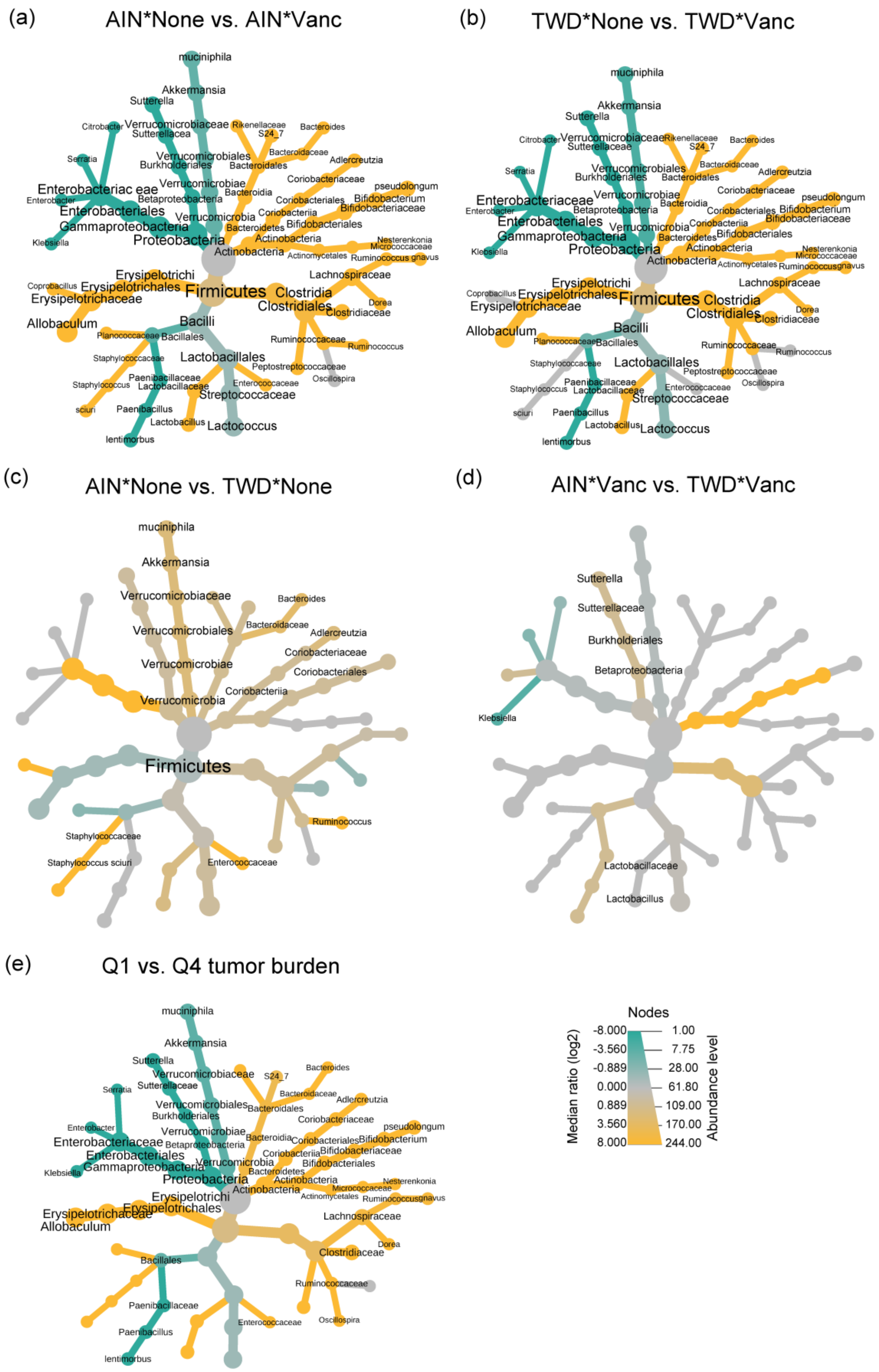
(a)
AIN*None vs. AIN*Vanc
muciniphila
Akkermansia
Sutterella
Citrobacter
Verrucomicrobiaceae
Rikenellaceae
S24_7
Bacteroides
Sutterellacea
Serratia
Verrucomicrobiales
Bacteroidaceae
Burkholderiales
Bacteroidales
Adlercreutzia
Coriobacteriaceae
Verrucomicrobiae
pseudolongum
Enterobacteriac eae
Bacteroidia
Enterobacter
Betaproteobacteria
Coriobacteriales
Bifidobacterium
Enterobacteriales
Bifidobacteriaceae
Verrucomicrobia
Coriobacteriia
Gammaproteobacteria
Bacteroidetes
Bifidobacteriales
Klebsiella
Proteobacteria
Actinobacteria
Nesterenkonia
Micrococcaceae
Actinobacteria
Actinomycetales
Ruminococcus gnavus
Erysipelotrichi
Lachnospiraceae
Coprobacillus
Erysipelotrichales
Firmicutes
Clostridia
Erysipelotrichaceae
Clostridiales
Dorea
Clostridiaceae
Allobaculum
Bacilli
Planococcaceae
Bacillales
Ruminococcaceae
Ruminococcus
Staphylococcaceae
Lactobacillales
Peptostreptococcaceae
Paenibacillaceae
Oscillospira
Staphylococcus
Lactobacillaceae
Enterococcaceae
Streptococcaceae
sciuri
Paenibacillus
Lactobacillus
Lactococcus
lentimorbus
(b)
TWD*None vs. TWD*Vanc
muciniphila
Akkermansia
Sutterella
Citrobacter
Verrucomicrobiaceae
Rikenellaceae
Bacteroides
Sutterellaceae
S24_7
Serratia
Verrucomicrobiales
Bacteroidaceae
Burkholderiales
Adlercreutzia
Bacteroidales
Coriobacteriaceae
Enterobacteriaceae
Verrucomicrobiae
pseudolongum
Bacteroidia
Enterobacter
Betaproteobacteria
Coriobacteriales
Bifidobacterium
Enterobacteriales
Bifidobacteriaceae
Verrucomicrobia
Coriobacteriia
Gammaproteobacteria
Bifidobacteriales
Bacteroidetes
Klebsiella
Proteobacteria
Actinobacteria
Nesterenkonia
Micrococcaceae
Actinobacteria
Actinomycetales
Ruminococcusgnavus
Erysipelotrichi
Lachnospiraceae
Coprobacillus
Erysipelotrichales
Firmicutes
Clostridia
Erysipelotrichaceae
Clostridiales
Dorea
Clostridiaceae
Allobaculum
Bacilli
Planococcaceae
Bacillales
Ruminococcaceae
Ruminococcus
Staphylococcaceae
Lactobacillales
Peptostreptococcaceae
Paenibacillaceae
Oscillospira
Staphylococcus
Lactobacillaceae
Enterococcaceae
Streptococcaceae
sciuri
Paenibacillus
Lactobacillus
Lactococcus
lentimorbus
(c)
AIN*None vs. TWD*None
muciniphila
Akkermansia
Verrucomicrobiaceae
Bacteroides
Bacteroidaceae
Verrucomicrobiales
Adlercreutzia
Coriobacteriaceae
Verrucomicrobiae
Coriobacteriales
Coriobacteriia
Verrucomicrobia
Firmicutes
Ruminococcus
Staphylococcaceae
Enterococcaceae
Staphylococcus sciuri
(d)
AIN*Vanc vs. TWD*Vanc
Sutterella
Sutterellaceae
Burkholderiales
Betaproteobacteria
Klebsiella
Lactobacillaceae
Lactobacillus
(e)
Q1 vs. Q4 tumor burden
muciniphila
Akkermansia
Sutterella
Verrucomicrobiaceae
S24_7
Bacteroides
Serratia
Sutterellaceae
Bacteroidaceae
Adlercreutzia
Verrucomicrobiales
Bacteroidales
Burkholderiales
Coriobacteriaceae
Verrucomicrobiae
pseudolongum
Enterobacter
Bacteroidia
Enterobacteriaceae
Betaproteobacteria
Coriobacteriales
Bifidobacterium
Enterobacteriales
Coriobacteriia
Bifidobacteriaceae
Klebsiella
Gammaproteobacteria
Verrucomicrobia
Bifidobacteriales
Bacteroidetes
Proteobacteria
Actinobacteria
Nesterenkonia
Actinobacteria
Micrococcaceae
Erysipelotrichi
Actinomycetales
Ruminococcusgnavus
Erysipelotrichaceae
Erysipelotrichales
Allobaculum
Lachnospiraceae
Dorea
Clostridiaceae
Bacillales
Ruminococcaceae
Paenibacillaceae
Enterococcaceae
Oscillospira
Paenibacillus
lentimorbus
Nodes
Median ratio (log2)
-8.000
-3.560
-0.889
0.000
0.889
3.560
8.000
Abundance level
1.00
7.75
28.00
61.80
109.00
170.00
244.00

Figure S5. (preceding page) Phylogenetic heat trees for selected comparisons among experimental groups. The heat tree analysis leverages the hierarchical structure of taxonomic classifications to quantitatively (using the median abundance) and statistically (using the non-parametric Wilcoxon Rank Sum test) depict taxonomic differences between microbial communities. Comparisons are (diet*antibiotic): (**a**), AIN*none vs. AIN*vanc, (**b**), TWD*none vs. TWD*vanc, (**c**) AIN*none vs. TWD*none, (**d**) and AIN*vanc vs. TWD*vanc. Also shown is the heat tree comparing low tumor burden (Q1) versus high tumor burden (Q4) (**e**). Tree nodes and branches are colored by the relative abundance level, with colors toward yellow indicating enrichment and colors toward green indicating depletion in the comparator group with respect to the reference (e.g., for panel a, yellow indicates enrichment of species *Akkermansia muciniphila* in the AIN*none group compared to the TWD*none group.) Nodes without labels were not significantly different by this nonparametric test. Abbreviations: AIN, the AIN93G diet; TWD, the total Western diet; Vanc, vancomycin.

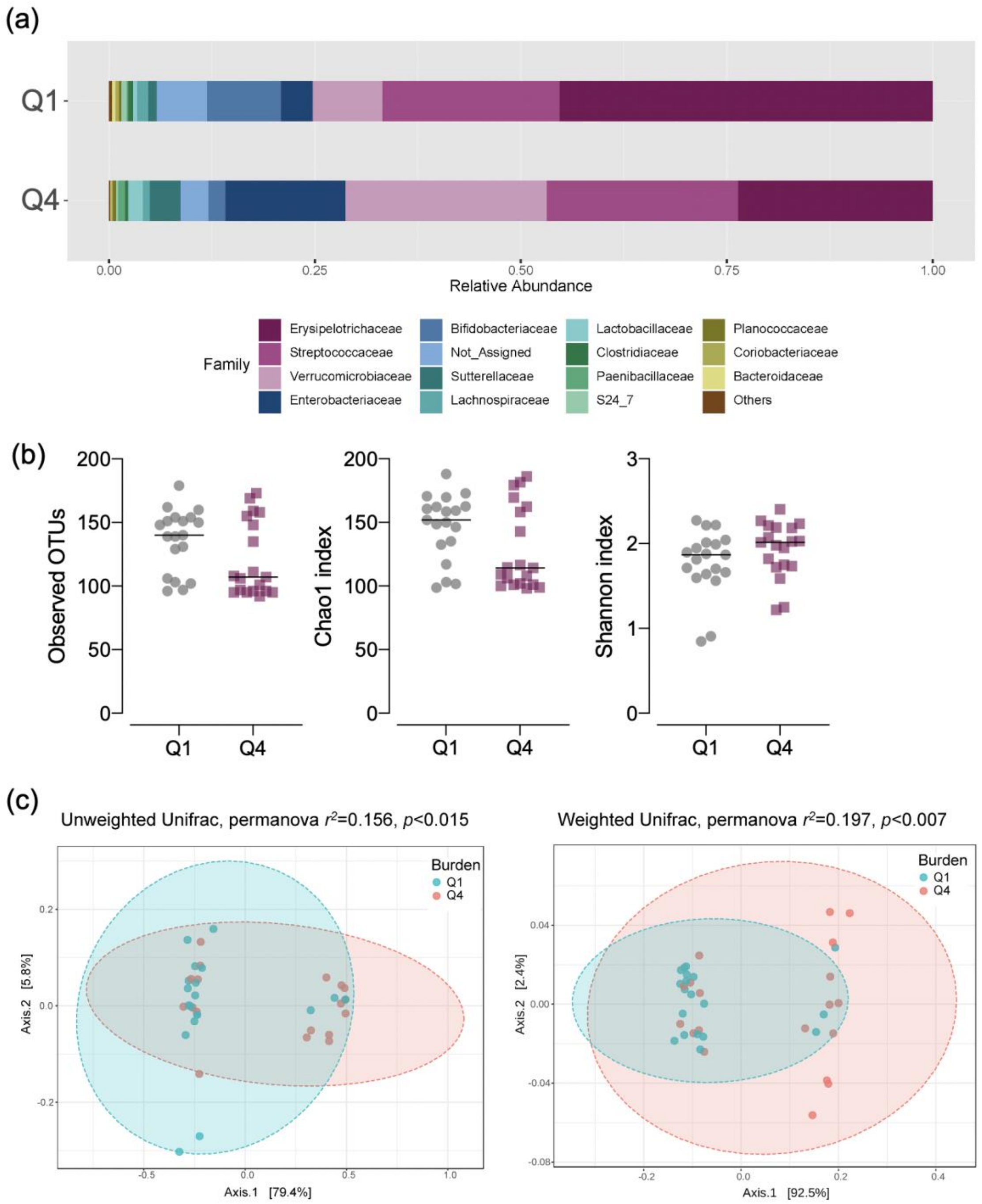

Figure S6. Taxonomic classification and diversity analyses for high and low tumor burden. Data were stratified into quartiles by tumor burden and the lowest 25% (Q1) and highest 25% (Q4) were compared. (**a**) Taxonomic classification at the family level. (**b**) Observed OTUs, Chao1 index, and Shannon index measures of alpha diversity. No significant differences between Q1 and Q4 were identified. (**c**) PCoA plots showing unweighted and weighted unifrac distances for beta diversity.

Table S1. Composition of experimental diets

**Nutrient**	**AIN93G**	**TWD**
Energy Density (kcal/g)	3.8	4.4
**Macronutrients**		
Carbohydrates (g/kg diet)		
Cellulose	50	30
Corn Starch	398	230
Maltodextrin	132	70
Sucrose	100	261
kcal (% of total)	*60.1%*	*54.5%*
Proteins (g/kg)		
Casein	200	190
L-cysteine	3	2.8
kcal (% of total)	*18.8%*	*15.4%*
Fats (g/kg)		
Anhydrous milk fat		36.3
Beef tallow		24.8
Cholesterol		0.4
Corn Oil		16.5
Lard		28
Olive oil		28
Soybean oil	70	31.4
kcal (% of total)	*17.2%*	*34.5%*
**Micronutrients**		
Minerals (mg/kg)		
Calcium	5000	2011
Copper	6	2.6
Iron	35	31
Magnesium	507	589
Phosphorus	3000	2757
Potassium	3600	5333
Selenium	0.15	0.2
Sodium	1019	7078
Zinc	30	25
Vitamins (unit/kg)		
Choline (mg)	1027	648
Folate (mg)	2	1.3
Niacin (mg)	30	50.6
Pyridoxine (mg)	6	3.9
Riboflavin (mg)	6	4.4
Thiamin (mg)	5	3.5
Vitamin A (IU)	4000	4300
Vitamin B12 (μg)	25	11
Vitamin D (IU)	1000	391
Vitamin E (IU)	75	24.6
Vitamin K (μg)	750	189

Notes: Composition of the TWD was previously published.[1] NHANES data do not provide intakes of biotin, chloride, iodine, manganese, pantothenic acid, or ultra-trace minerals. The levels of these micronutrients in the AIN93G diet were used in the formulation of the TWD. Abbreviations: AIN, American institute of Nutrition; TWD, total Western diet

[1] Hintze, K.J.; Benninghoff, A.D.; Ward, R.E. Formulation of the total western diet (TWD) as a basal diet for rodent cancer studies. *J. Agric. Food Chem.* **2012**, *60*, 6736-6742, doi:10.1021/jf204509a.

Table S2. MetagenomeSeq analyses of differential abundance of family- and genus-level taxa by experimental group and tumor burden

Family, Genus, Species	Diet main effect	AB main effect	Pairwise comparisons for diet and antibiotic treatment				High (Q4) vs. Low (Q1) Tumor Burden
			AIN: None vs. Vancomycin	TWD: None vs. Vancomycin	None: AIN vs. TWD	Vancomycin: AIN vs. TWD	
**Actinobacteria**	1.07E-01	**5.61E-14**	1.84E-04	6.35E-10	4.58E-01	1.00E+00	1.59E-02
Bifidobacteriaceae	8.36E-01	**6.98E-27**	**1.87E-15**	**5.74E-13**	3.91E-01	1.00E+00	6.41E-02
Bifidobacterium	7.13E-01	**5.31E-29**	**1.67E-14**	**5.24E-16**	6.08E-01	1.00E+00	**4.08E-02**
Coriobacteriaceae	5.88E-03	**9.45E-28**	**2.58E-15**	**4.47E-17**	**4.68E-02**	1.00E+00	4.95E-01
Adlercreutzia	1.00E-03	**2.45E-19**	**1.46E-13**	**1.70E-09**	**2.91E-04**	1.00E+00	4.34E-01
Micrococcaceae	9.24E-01	**2.55E-06**	**3.50E-04**	**9.33E-04**	9.43E-01	1.00E+00	6.34E-02
Nesterenkonia	8.77E-01	**3.36E-07**	**4.63E-04**	**6.82E-04**	7.54E-01	1.00E+00	7.63E-02
**Bacteroidetes**	1.07E-01	**1.08E-09**	1.91E-07	2.80E-04	9.14E-01	1.00E+00	7.22E-02
Bacteroidaceae	7.56E-02	**2.18E-12**	**5.79E-10**	**1.08E-06**	1.90E-01	1.00E+00	1.78E-01
Bacteroides	1.20E-01	**4.65E-13**	**1.73E-07**	**2.90E-07**	1.50E-01	1.00E+00	1.75E-01
Bacteroidales S24_7	1.00E+00	1.00E+00	**3.94E-09**	**3.95E-09**	9.00E-01	1.00E+00	6.41E-02
Rikenellaceae	2.22E-01	**3.58E-07**	**7.65E-06**	**1.50E-04**	2.11E-01	1.00E+00	2.06E-01
**Firmicutes**	4.18E-01	**2.55E-16**	3.81E-05	1.11E-09	4.58E-01	9.28E-01	1.95E-01
Clostridiaceae	9.24E-01	**3.77E-17**	**5.36E-11**	**3.62E-09**	5.53E-01	1.00E+00	7.90E-01
Christensenellaceae	1.00E+00	1.00E+00	1.00E+00	1.00E+00	2.43E-01	1.00E+00	
Enterococcaceae	2.60E-04	**3.41E-06**	**1.88E-05**	1.00E+00	**7.34E-03**	1.00E+00	6.30E-02
Erysipelotrichaceae	9.96E-01	**1.87E-52**	**1.35E-25**	**2.27E-32**	6.54E-01	2.42E-01	1.78E-01
Allobaculum	7.13E-01	**5.01E-52**	**6.80E-21**	**8.30E-28**	5.87E-01	3.11E-01	1.83E-01
Coprobacillus	1.28E-01	**2.46E-12**	**3.08E-05**	1.00E+00	2.65E-01	1.00E+00	
Lachnospiraceae	2.22E-01	**1.59E-26**	**3.41E-18**	**9.19E-15**	4.15E-01	1.00E+00	1.78E-01
Dorea	7.51E-02	**8.07E-10**	**7.59E-06**	**5.02E-06**	8.73E-02	1.00E+00	9.04E-01
Lactobacillaceae	9.75E-01	**6.50E-10**	**2.99E-06**	**1.20E-07**	8.30E-01	**5.85E-03**	4.95E-01
Lactobacillus	9.11E-01	**4.22E-12**	**5.65E-06**	**1.88E-07**	7.54E-01	1.00E+00	5.42E-01
Leuconostocaceae	1.00E+00	1.00E+00	1.00E+00	**4.45E-05**	2.11E-01	1.00E+00	
Paenibacillaceae	6.11E-01	**1.21E-27**	**1.17E-13**	**6.12E-16**	1.00E+00	4.04E-01	6.41E-02
Paenibacillus	9.11E-01	**4.97E-30**	**1.67E-14**	**4.88E-16**	1.00E+00	9.67E-01	**4.08E-02**
Peptostreptococcaceae	2.22E-01	**4.87E-14**	**4.70E-09**	**1.36E-07**	9.84E-01	1.00E+00	7.90E-01
Planococcaceae	9.75E-01	**1.77E-08**	**1.09E-05**	**3.38E-07**	3.26E-01	1.00E+00	3.82E-01

Table S2. MetagenomeSeq analyses of differential abundance of family- and genus-level taxa by experimental group and tumor burden

Family, Genus, Species	Diet main effect	AB main effect	Pairwise comparisons for diet and antibiotic treatment: AIN: None vs. Vancomycin	TWD: None vs. Vancomycin	None: AIN vs. TWD	Vancomycin: AIN vs. TWD	High (Q4) vs. Low (Q1) Tumor Burden
Ruminococcaceae	2.22E-01	**7.22E-12**	**1.29E-06**	**4.75E-06**	9.84E-01	1.00E+00	1.05E-01
Oscillospira	1.20E-01	**8.24E-07**	**7.69E-04**	**1.64E-05**	5.87E-01	1.00E+00	4.34E-01
Ruminococcus	3.86E-01	**1.21E-23**	**1.06E-12**	**2.91E-10**	6.08E-01	1.00E+00	1.83E-01
Staphylococcaceae	2.22E-01	**3.39E-09**	**5.38E-06**	**1.15E-04**	2.11E-01	1.00E+00	5.70E-01
Staphylococcus	1.70E-01	**5.97E-09**	**1.29E-05**	**1.27E-04**	**1.45E-02**	1.00E+00	6.32E-01
Streptococcaceae	2.22E-01	**1.14E-02**	8.45E-02	**1.32E-02**	2.52E-01	4.70E-01	7.91E-01
Lactococcus	1.35E-01	**2.19E-03**	6.24E-02	**2.15E-02**	1.76E-01	6.05E-01	8.24E-01
**Proteobacteria**	1.07E-01	**4.47E-17**	3.03E-05	2.63E-10	1.28E-02	9.28E-01	5.52E-02
Enterobacteriaceae	8.25E-01	**2.83E-56**	**1.30E-26**	**2.98E-27**	1.00E+00	4.70E-01	1.78E-01
Citrobacter	7.13E-01	**2.42E-09**	**3.08E-05**	**1.62E-07**	1.00E+00	1.67E-01	
Enterobacter	7.51E-02	**4.93E-15**	**7.03E-06**	**1.07E-12**	1.00E+00	4.21E-01	1.63E-01
Klebsiella	3.06E-02	**4.27E-10**	1.00E+00	**2.79E-06**	1.00E+00	**4.55E-02**	**4.08E-02**
Serratia	9.11E-01	**9.25E-19**	**1.86E-07**	**3.88E-12**	1.00E+00	3.99E-01	1.83E-01
Pseudomonadaceae	1.00E+00	1.00E+00	1.00E+00	**1.59E-05**	1.00E+00	8.86E-02	
Pseudomonas	1.00E+00	1.00E+00	1.00E+00	**2.73E-04**	1.00E+00	**4.61E-02**	
Sutterellaceae	9.75E-01	**1.18E-32**	**1.79E-21**	**1.66E-16**	3.29E-01	2.42E-01	1.78E-01
Sutterella [a]	4.17E-01	**1.00E-35**	**5.08E-19**	**4.88E-16**	7.21E-01	**4.55E-02**	**1.63E-01**
**Teneriutes**	1.00E+00	1.00E+00	**7.26E-04**	1.00E+00	1.28E-02	1.00E+00	
**Verrucomicrobia**	3.24E-01	2.89E-01	**1.75E-03**	3.11E-01	7.00E-01	9.28E-01	1.32E-01
Verrucomicrobiaceae	5.17E-01	**5.05E-09**	**8.57E-05**	**1.36E-07**	4.15E-01	9.52E-01	2.06E-01
Akkermansia	2.27E-01	**1.23E-10**	**1.23E-04**	**2.94E-06**	5.53E-01	6.05E-01	1.77E-01

Values above are the FDR-corrected p-values obtained using Microbiome Analyst metagenomeSeq with alpha = 0.05 and the zero-inflated gaussian fit (n=17 to 23). Abbreviations: AIN, the AIN93G diet; TWD, the total Western diet; AB, antibiotic; Q1, lowest 25% quartile; Q4, highest 25% quartile. Empty cells indicate taxa was not included in the metagenomeSeq report for that specific comparison due to low counts.

[a] The *Sutterella* genus was classified incorrectly as belonging to family Alcaligenaceae by GreenGenes database. The correct classification is shown here
